# Supplementary material for: A Simple Validated Method for the Estimation of Pepsin Activity in Microtiter Array for the INFOGEST Protocol
Source: Foods. 2023 Oct 20;12(20):3851. doi: 10.3390/foods12203851 (PMC10606326; doi:10.3390/foods12203851)
Supplement: Supplementary file 1 [file foods-12-03851-s001.zip › Supplementary material s1 - Estimation of pepsin activity via the Folin Ciocalteu method.pdf]

Supplementary material S1:

Estimation of pepsin activity via the Folin–Ciocalteu method

This supp data shows the detailed steps for pepsin activity estimation: (I) Preparation of reagents and (II) digestion procedure the (III) analysis through the Folin-Ciocalteu method in microtiter and (IV) pepsin activity calculation.

I. **Preparation of reagents**

1. 300 mM sodium chloride, 20 mM Tris hydrochloride HCl solution (NaCl/Tris-HCl).
2. 300 mM hydrochloric acid HCl solution.
3. 10 mM hydrochloric acid HCl solution.
4. 6 % sodium carbonate (Na_2CO_3) solution.
5. 2% haemoglobin stock solution.

Weight 5 g of haemoglobin and complete to 20 mL purified water, then acidify with 2.5 mL of 300 mM HCl, adjust pH at 2 with NaOH 1 M and complete with water to a volume of 25 mL. Keep refrigerated.

6. 0,5 mg/mL pepsin solution

Weight 12.5 mg of pepsin, dilute it with 12.5 mL of de NaCl/Tris HCl solution previously prepared. Adjust to pH 6.5 with sequential addition of 10 µL of NaOH 1 M solution, complete to 25 mL with deionized water.

7. 5 % w/v trichloroacetic acid (TCA)

Weight 2.5 g of TCA and complete to 50 mL with deionized water.

8. 500 µg/mL L-Tyrosine (L-Tyr) stock solution

Weight 12.5 mg of the standard of high purity and dissolve with 10 mM HCl. Shake vigorously and apply ultrasonic if necessary until complete dissolution. Do triplicate whenever possible.

9. L-Tyrosine standard curve

Prepare the standard curve in microcentrifuge tubes, using 10 mM HCl as solvent.

N° Solution	L-Tyr concentration (µL/mg)	L-Tyr stock aliquot (µL)	10 mM HCl volume completion (µL)	**Concentration levels (mM)**
1	0	0	1000	**0.000**
2	5	10	990	**0.028**
3	10	20	980	**0.055**
4	25	50	950	**0.138**
5	40	80	920	**0.221**
6	55	110	890	**0.304**

II. Digestion procedure

1. Pepsin solutions

Prepare six pepsin solutions in microcentrifuge tubes with the 0.5 mg/mL stock solution. These must be maintained over ice to avoid enzyme activation.

Pepsin solutions (µg/mL)	Stock solution aliquot (µL)	Volume of 10 mM HCl (µL)
5	100	900
10	200	800
15	300	700
20	400	600
25	500	500
30	600	400

2. Digestion of haemoglobin

For the digestion six concentrations of pepsin are tested, which are divided in 6 for the tests and 6 for the blanks. The protocol continues as follows:

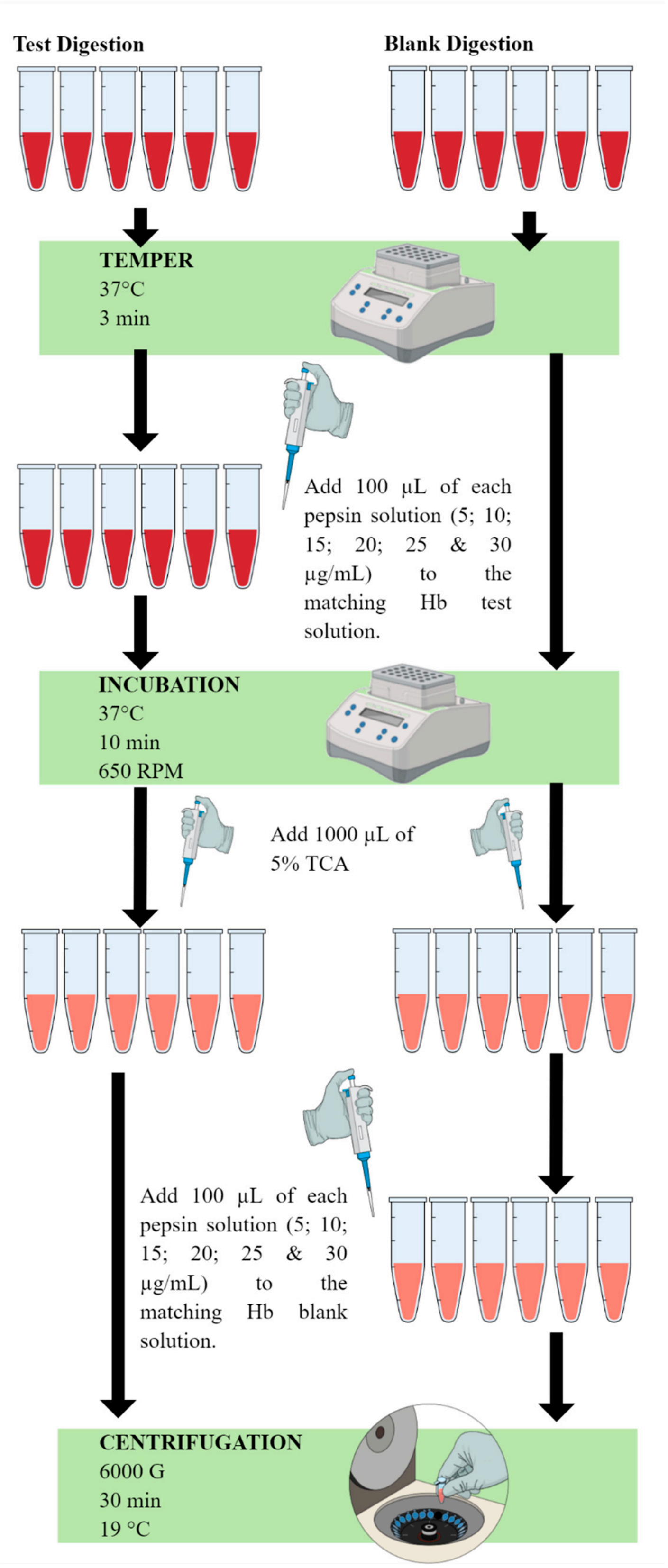

First, add 500 µL of haemoglobin to each of the twelve microcentrifuge tubes and temper them in the incubator for 3 min at 37 °C.

Add 100 µL of the pepsin solutions to the test labeled tubes. Rapidly incubate the test and blank tubes for 10 min at 37 °C and 650 RPM.

Past the 10 min, briefly add 1000 µL of 5% TCA to each of the twelve solutions. Next add 100 µL of the pepsin solutions to the matching blank labeled tubes.

The twelve tubes are centrifuged at 6000 g for 30 min at room temperature, 19 °C. With a micropipette, collect the supernatant in different microcentrifuge tubes, being careful to not take the precipitate.

III. Analysis through the Folin Ciocalteu method

In a 96-well transparent microtiter add 50 µL of the collected supernatant, 50 µL of 20% Folin Ciocalteu reagent (prepared at the moment) and 100 µL of 6% sodium carbonate to each used well. The absorbance of each well is measured at 760 nm after 10 min of incubation in darkness at 37 °C. It is recommended to adjust the pathlength correction with 977 nm to 1 cm, for the optical length normalization.

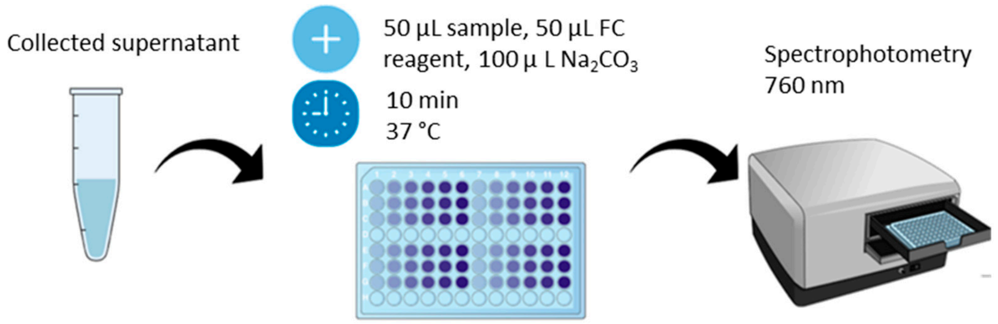

Register the absorbance of test and blanks solutions and graph the difference between them. Consider only results in the linear response. Also include the L-Tyr standard curve previously prepared.

IV. Calculation of activity for the INFOGEST protocol

1. Determine the L-Tyr concentration in both Test and Blank supernatants by interpolation of the L-Tyr standard curve.
2. Graph the difference between L-Tyr concentracion (µmol/mL) of matching Test and Blank supernatant solutions. Remove nonlinear results.
3. Complete the calculation by using the next equation, which is automatized in the attached excel file.

$$units/mg = \frac{[L\ Tyr]_{Test} - [L\ Tyr]_{blank}}{\Delta t \times X \times 0.0125}$$

[L Tyr]: L-Tyrosine concentration in the test and blank supernatant solutions in mM determined by the linear equation of the standard.
Δt: Time of reaction, generally 10 minutes.
X: Concentration of pepsin powder in the final reaction mixture in mg/mL.
0.0125: Absorbance value attributed to the activity of one unit of pepsin.

The final result is expressed with a 95% confidence interval.
